# Housing Characteristics and their Influence on Health-Related Quality of Life in Persons Living with HIV in Ontario, Canada: Results from the Positive Spaces, Healthy Places Study

**DOI:** 10.1007/s10461-012-0284-0

**Published:** 2012-08-18

**Authors:** Sean B. Rourke, Tsegaye Bekele, Ruthann Tucker, Saara Greene, Michael Sobota, Jay Koornstra, LaVerne Monette, Jean Bacon, Shafi Bhuiyan, Sergio Rueda, James Watson, Stephen W. Hwang, James Dunn, Keith Hambly, The Positive Spaces Healthy Places Team

**Affiliations:** 1 The Ontario HIV Treatment Network, 600-1300 Yonge St, Toronto, ON M4T 1X3 Canada; 2 Centre for Research on Inner City Health, The Keenan Research Centre, Li Ka Shing Knowledge Institute, St. Michael’s Hospital, Toronto, Canada; 3 Department of Psychiatry, University of Toronto, Toronto, Canada; 4 The CIHR Centre for REACH in HIV/AIDS (Research Evidence into Action for Community Health), Toronto, Canada; 5 Hamilton AIDS Network, Hamilton, Canada; 6 Faculty of Social Sciences, School of Social Work, McMaster University, Hamilton, Canada; 7 Fife House, Toronto, Canada; 8 AIDS Thunder Bay, Thunder Bay, Canada; 9 Bruce House, Ottawa, Canada; 10 Ontario Aboriginal HIV/AIDS Strategy, Toronto, Canada; 11 Faculty of Medicine, University of Toronto, Toronto, Canada; 12 Department of Health, Aging & Society, McMaster University, Hamilton, Canada

**Keywords:** Housing, Housing affordability, Housing satisfaction, Health-related quality of life, HIV

## Abstract

Although lack of housing is linked with adverse health outcomes, little is known about the impacts of the qualitative aspects of housing on health. This study examined the association between structural elements of housing, housing affordability, housing satisfaction and health-related quality of life over a 1-year period. Participants were 509 individuals living with HIV in Ontario, Canada. Regression analyses were conducted to examine relationships between housing variables and physical and mental health-related quality of life. We found significant cross-sectional associations between housing and neighborhood variables—including place of residence, housing affordability, housing stability, and satisfaction with material, meaningful and spatial dimensions of housing—and both physical and mental health-related quality of life. Our analyses also revealed longitudinal associations between housing and neighborhood variables and health-related quality of life. Interventions that enhance housing affordability and housing satisfaction may help improve health-related quality of life of people living with HIV.

## Resumen

A pesar de que la falta de vivienda está vinculada a consecuencias adversas para la salud, conocemos poco sobre el impacto de los aspectos cualitativos de la vivienda sobre la salud. Este estudio examinó la asociación entre elementos estructurales de la vivienda, accesibilidad a la vivienda, satisfacción con la vivienda y la calidad de vida relacionada con la salud (CVRS) por un período de un año. Los participantes fueron 509 personas que viven con el VIH en Ontario, Canada. Análisis de regresión lineal multivariado se llevaron a cabo para examinar la relación entre las variables de vivienda y la calidad de vida relacionada a la salud física y mental. Los resultados de los análisis transversales mostraron una asociación significativa entre las variables de la vivienda y del vecindario - incluyendo lugar de residencia, accesibilidad de precio, la estabilidad de la vivienda y la satisfacción con las dimensiones materiales, de significado y espaciales de la vivienda - y la calidad de vida tanto física como mental. Nuestros análisis también revelaron asociaciones longitudinales entre las variables de vivienda y del vecindario con la CVRS a traves del tiempo. Las intervenciones que mejoran el acceso y la satisfacción con la vivienda pueden ayudar a mejorar la CVRS de las personas que viven con el VIH.

## Introduction

Housing is one of the major determinants of health—it is a medium through which socio-economic status is expressed and health determinants operate [1, 2]. Housing can be conceptualized as an intermediate structural factor that links broader societal processes and influences with an individual’s immediate social and physical environment [3]. It provides physical security and protection from the elements, and plays a central role in determining an individual’s physical and social risk environment [4, 5]. Housing can also provide a source of identity and belonging [3, 4, 6], and create a physical or social space in which social ties and positive social relations are fostered and maintained [3, 6].

Housing research has identified three main dimensions of housing that are relevant to health: material, meaningful and spatial dimensions [7]. The material dimension of housing refers to: the direct physical and structural aspects, which confer a protected space and facilities for maintaining physical well-being (e.g., to sleep, wash, prepare food); and the physical integrity of the home including the state of repair and housing cost, which is an important factor as higher housing cost relative to income may eventually result in homelessness [8, 9]. The meaningful dimension of housing refers to the social meanings that people commonly attach to housing including sense of belonging and control in the home. Experiencing a ‘‘sense of home’’ contributes to ontological security—a sense of order, continuity and meaning with regard to an individual’s experiences [10, 11]—which may lead to a sense of personal and social identity that helps build resistance to risky behaviors [3]. The spatial dimension of housing refers to the location of housing relative to services and facilities needed to sustain life and health. As an “individual’s home is considered as a crucial locus for everyday life” [7], its location relative to services and amenities needed for healthful everyday life is a crucial pathway through which housing may affect health.

Housing occupies an important place in the causal chains linking poverty and inequality, HIV risk, and outcomes of HIV infection [3]. Homelessness or unstable housing is linked with elevated rates of HIV infection [12–15], mediated through behaviors associated with HIV risks such as injection drug use and needle sharing, multiple sex partners, unprotected sex with casual partners, and exchange of sex for money, food, drugs or shelter [12, 13, 16–19]. Housing can also play an important intermediary role in HIV prevention and care. Homelessness reduces the effectiveness of HIV risk reduction programs [20]. People who are homeless or unstably housed have lower levels of health care utilization and adherence to antiretroviral treatment than those with stable housing [21–25]. A growing body of research has also documented associations between lack of stable and adequate housing and various health outcomes including hepatitis C, pneumonia, tuberculosis, anxiety, depression, poorer self-rated health, and mortality [25–28]. On the other hand, there is some evidence to show that housing interventions for the homeless can improve health outcomes [29].

Despite the strong evidence linking lack of housing (i.e., homelessness) and unstable housing with health status, there is a gap in the literature on the impact of housing affordability on health. Among people living with HIV, there is a great need for affordable housing and rental assistance [10, 30, 31], as their ability to meet housing costs is affected by the high levels of unemployment and poverty associated with the disease [32]. Difficulty meeting housing costs is associated with higher risk of losing housing [31] and may lead to higher levels of anxiety and stress. People facing difficulty meeting housing costs can be trapped in inappropriate and unsatisfying housing. Housing cost can also compromise one’s ability to spend on other health-enhancing goods and services [7].

Research on the general population indicates an association between higher percentage of household income spent on housing and poor health status [33]. Preliminary research evidence in the general population suggests that meaningful dimensions of housing may play a role in maintaining health and healthy behaviour. For example, Dunn and Hayes [34] found that the meaning people invest in their homes, their satisfaction with their homes, and the amount of control they were able to exercise in the social and economic aspects of their domestic relations were associated with self-reported general health and mental health. Spatial attributes of places or neighborhoods may contribute to health status independent of characteristics of individual residents [35]. The location of home in relation to health and other services and amenities required to sustain life can affect health outcomes. Characteristics of neighborhoods also may affect one’s social norms and social norms in turn affect health behaviours. Results from a cross-sectional study, for example, indicate that individuals who agreed that their home is a good place to live their life were more likely to report better mental health [34]. The spatial dimension of one’s residence, therefore, can be a pathway through which housing may affect health [7]. In the context of HIV, however, there is a gap in the literature on the effects of meaningful and spatial attributes of housing on health outcomes.

**Fig. 1:**
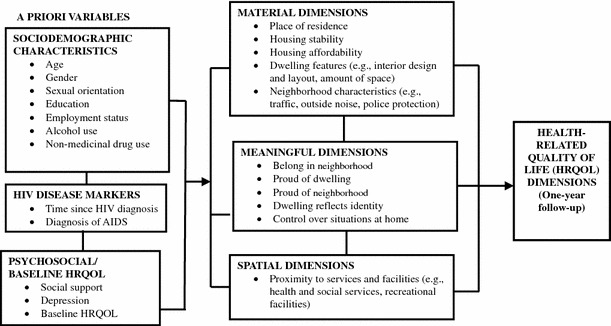
Analytical model for housing and HRQOL

The primary objective of this study is to examine the relationship between material, meaningful and spatial dimensions of housing and health-related quality of life (HRQOL = health-related quality of life) among adults living with HIV. Using data collected at two time points (baseline and 1-year follow-up) and an adopted analytical model (see Fig. 1) [34], we will examine whether higher satisfaction with housing and neighborhood attributes are associated with better physical and mental HRQOL. We will also assess whether housing and neighborhood attributes predict improvement in HRQOL over a 1-year period of time. We hypothesize that a higher level of satisfaction with housing and neighborhood dimensions (i.e., material, meaningful and spatial) would be associated with better HRQOL and lead to improvements in HRQOL.

## Methods

### Study Sample and Recruitment

We used baseline and 1-year follow-up data from the CIHR-funded *Positive Spaces, Healthy Places* (PSHP) study. PSHP is an observational cohort of 602 adults living with HIV in Ontario, Canada designed to evaluate the health effects of housing. Participants were recruited through community-based AIDS service organizations and were eligible if they were HIV-positive adults (18 years or older) living in Ontario and able to provide informed consent. To achieve as representative a sample as possible, the recruitment strategy used a wide range of access points throughout the province, including: homeless shelters; agencies serving women, families, and youth; Aboriginal organizations; transitional housing providers; and supportive housing agencies. Efforts were made to include harder-to-reach populations such as injection drug users and street-involved communities (i.e., individuals who live in and out of hostels and homeless shelters). To minimize bias, sampling was stratified and recruitment targets were established that reflected the regional, gender, sexual orientation and ethnic distribution of the HIV prevalence in Ontario [36]. A post hoc power calculation showed the PSHP sample has a power of 0.90 to detect a medium (Cohen’s d = 0.5) to high (Cohen’s d = 0.8) effect size of change in HRQOL at an alpha level of 0.05.

The study surveys and questionnaires included comprehensive social and behavioural measures (taking 60–90 min to complete) and were administered in face-to-face interviews by trained peer research assistants—people living with HIV. Their role was an important element in the overall study design as it reflected the study team’s strong commitment to community-based research and the Greater and Meaningful Involvement of People Living with HIV Principles [37]. Ethics approval for this study was obtained from the Research Ethics Board of McMaster University (Hamilton, Canada), the University of Toronto (Toronto, Canada), and York University (Toronto, Canada). Participants were paid an honorarium of $60 and $40 for the baseline and 1-year follow-up interviews, respectively.

### Measures

We collected self-reported information on sociodemographic (e.g., age, gender, education employment, income), HIV disease markers (e.g., time since HIV diagnosis, diagnosis of AIDS), alcohol use [38], illicit drug use [39], and psychosocial variables including perceived social support [40] and depressive symptoms [41].

#### Housing Variables

The housing-related variables included place of residence in Ontario [i.e., living in the Greater Toronto Area (GTA = Greater Toronto Area) versus living outside of the GTA], difficulty paying housing cost (very difficult/fairly difficult versus a little difficult/not at all difficult), receipt of rent assistance (yes vs. no), and number of times moved in the past year (twice or more vs. once or less). Participants were also asked whether they were currently homeless or lived in inadequate housing (yes vs. no). For the purpose of this study, homelessness was defined as living in an emergency shelter, living in a car, living on the streets, or couch-surfing while inadequate housing was defined as living in a motel, hotel or boarding house. History of homelessness (at least once in my lifetime vs. never) and history of incarceration (yes, at least once in my lifetime vs. never) were also assessed. Participants were also asked if they have ever experienced discrimination when trying to get housing services (yes, at least once in my life time vs. never) and the potential subjective reasons associated with this experience. We also assess participants’ level of satisfaction with their dwelling (10 items), satisfaction with their neighborhood (8 items) and meaningful dimension of their dwelling and neighborhood (8 items) using a 26-item instrument (rated on a 5-point Likert scale) that was adopted from another study [34].

To isolate the key dimensions of housing satisfaction used in the regression analyses, we subjected the 26 items assessing housing satisfaction to a principal component analysis (PCA) with varimax rotation after reverse coding items so that higher scores indicate higher degree of satisfaction or meaning of dwelling or neighborhood aspects. Data on these items were missing for 34 individuals who were homeless or had inadequate housing at baseline and were substituted with the mean values of the entire sample. After examining the factor loadings on a preliminary analysis, we removed two items due to low communality (<0.40) and one item due to high cross-loading (>0.40) on two factors. The PCA analysis was repeated with the remaining 23 items and yielded a 4-factor solution. More specifically, the four dimensions consisted of;

1. ‘dwelling features’ factor which had 7 items assessing satisfaction with space (e.g., amount of space), light (e.g., exposure to sunlight), in-door heating (e.g., heating) and air quality (e.g., in-door air quality);
2. ‘neighborhood characteristics’ factor that included 7 items related to the physical (e.g., parks and green space), noise (e.g., noise from outside the building), and safety (e.g., safety and security of building) features of the neighborhood;
3. ‘meaning of dwelling and neighborhood’ factor including 6 items related to identity (e.g., dwelling is a good reflection of who I am), status (i.e., proud of dwelling, proud of neighborhood), control (e.g., at home, I have control over most situations), and sense of belonging (e.g., belong in my neighborhood); and
4. ‘proximity to services and facilities’ factor including 3 items associated with location of dwelling relative to services (e.g., accessibility to health and social services) and facilities (e.g., accessibility to recreational facilities).

Internal consistency was acceptable for all factors: dwelling features, α = 0.87; neighborhood characteristics features, α = 0.87; meaning of dwelling and neighborhood, α = 0.86; and proximity to services and facilities, α = 0.72. Raw scores for each factor solution were then computed by summing items in each factor and used in descriptive statistics. For the multivariate regression analyses, however, we used regression factor scores from the PCA to minimize the potential harmful effects of collinearity.

##### Health-Related Quality of Life

Participants’ HRQOL was assessed using the medical outcomes study HIV (MOS-HIV) survey, a 35-item HIV-specific quality of life tool [42]. It measures general health perceptions (5 items), physical functioning (6 items), social functioning (2 items), role functioning (2 items), cognitive functioning (4 items), pain (2 items), mental health (5 items), energy/fatigue (4 items), health distress (4 items), and quality of life (1 item). All scales were linearly transformed into a 0 (worst health) to 100 (best health) scale, and then were converted into z-scores to standardize the scores to the reference population of patients with HIV/AIDS [43]. Finally, we created two aggregate scores—physical health summary (PHS) and mental health summary (MHS)—following the developer’s instructions [42].

### Statistical Analyses

Descriptive statistics (frequency, mean, standard deviation) were obtained on all variables of interest. McNemar and paired student *t* tests were used to compare the housing and neighborhood variables and HRQOL outcomes at baseline and 1-year follow-up.

We then fitted cross-sectional and longitudinal multivariable linear regression models to examine the association between housing and neighborhood variables and HRQOL, adjusting for control variables. We selected those variables with the strongest association with the outcomes of interest in bivariate models. Variables were entered sequentially into the multivariable regression models in three different blocks. Sociodemographic and HIV disease variables were entered in the first block as they are considered important determinants of HRQOL. Baseline social support, depressive symptoms, substance use, and HRQOL variables were entered as the second block followed by housing and neighborhood variables as the third and final block. All continuous predictor variables were mean centered before they were entered into the multivariable regression models. Condition indices and variance proportions were computed to examine degrading or harmful multicollinearity among all the independent and controlling variables. Because of multicollineraity, depression was excluded from the final multivariate regression model for MHS. Missing data for age and time since HIV diagnosis were replaced with mean values. Statistical significance was set at *p* < 0.05 and all reported *p* values are two-tailed. All analyses were performed using SPSS 16.0 (SPSS Inc., Chicago, IL).

## Results

Of the 602 individuals enrolled at baseline, 93 (15 %) were lost over the 1-year follow-up. As a result, 509 individuals completed the 1-year follow-up and were included in the final analyses.^1^ Baseline sociodemographic, psychosocial, and health characteristics of the final sample are presented in Table 1. Participants were predominantly middle-aged, male, gay, lesbian, or bisexual, Caucasian, and unemployed. At baseline, participants have lived with HIV for an average of 11.5 years; half were diagnosed with at least one AIDS defining condition, and close to 75 % were on antiretroviral treatment. About 50 % used illicit drugs in the past 12 months.

**Table 1:** Sociodemographic, housing, HIV disease, and psychosocial characteristics of participants at baseline (*N* = 602)

Characteristics	*n* or mean	% or (SD)
Age (years)^a^	43.1	(8.6)
Gender		
Female or transgender	148	25 %
Male	454	75 %
Sexual orientation		
Gay, lesbian, or bisexual	374	62 %
Heterosexual	228	38 %
Race or ethnicity		
Caucasian	441	73 %
Non-Caucasian	161	27 %
Education		
< high school	133	22 %
≥ high school	469	78 %
Employment status		
Employed	121	20 %
Unemployed/retired/disabled	481	80 %
Personal income (per month)^*b*^		
≤ $1,200/month	342	57 %
≥ $1,200/month	225	37 %
Alcohol consumption (AUDIT-10 score)	3.4	(5.6)
Substance use (DAST-20 index)	4.0	(5.3)
Live in the greater Toronto area		
Yes	374	62 %
No	228	38 %
Homeless or live in inadequate housing (yes)^*a*^		
Yes	34	6 %
No	568	94 %
Moved twice or more in the past 12 months (yes)		
Yes	73	12 %
No	529	88 %
Receive rental assistance (yes)		
Yes	370	61 %
No	232	39 %
Experienced difficulty paying housing cost (yes)^*b*^		
Yes	236	39 %
No	366	61 %
Time since HIV diagnosis (years)^c^	11.2	(6.5)
On antiretroviral treatment		
Yes	446	74 %
No	156	26 %
Ever diagnosed with AIDS		
Yes	298	49 %
No	304	51 %
Depressive symptoms (CESD-R score)	17.9	(15.3)
Perceived social support (MOS-SSS score)	62.1	(18.9)

*SD* standard deviation

^a^Data missing for 33 individuals

^b^Data missing for 35 individuals

^c^Data missing for 3 individuals

Table 2 summarizes the housing and neighborhood characteristics of participants and HRQOL dimensions at baseline and 1-year follow-up time. The majority of the study participants lived in the GTA. Only 4 % at baseline and 2 % at 1-year follow-up were either homeless (i.e., living in the street, cars, and parks) or were living in significant and inadequate housing (i.e., hotels, motels, shelters, and couch-surfing). At baseline, 11 % reported that they moved twice or more in the past 12 months and a lower proportion (7 %) moved twice or more between baseline and 1-year follow-up. Nearly two-thirds (63 %) were receiving rental assistance at baseline. Difficulty meeting monthly housing cost among participants was high (44 %) at baseline and improved at 1-year follow-up (31 %). Satisfaction scores of ‘dwelling features’, ‘neighborhood characteristics’, ‘meaning of dwelling and neighborhood’, and ‘proximity to services and facilities’ also improved over the 1-year follow-up.

**Table 2:** Housing and neighborhood variables and HRQOL of participants at baseline and 1-year follow-up (*N* = 509)

Variables	Baseline		One-year follow-up		*p*
	*n* or mean	% or (SD)	*n* or mean	% or (SD)	
Housing and neighborhood variables					
Live in the greater Toronto area*	300	(59 %)	287	(56 %)	0.001
Homeless or live in inadequate housing (yes)^a^	19	(4 %)	12	(2 %)	0.265
Moved ≥2 in the past 12 months (yes)	56	(11 %)	34	(7 %)	0.008
Receive rental assistance (yes)	318	(62 %)	310	(61 %)	0.256
Experienced difficulty paying housing cost (yes)^b^	223	(44 %)	159	(31 %)	0.002
Housing and neighborhood satisfaction					
Dwelling features	21.0	(6.0)	21.5	(5.0)	0.015
Neighborhood characteristics	23.3	(6.5)	24.1	(5.7)	0.003
Meaning of dwelling and neighborhood	21.4	(6.2)	22.0	(5.4)	0.020
Proximity to services and facilities	9.9	(3.0)	10.2	(2.7)	0.046
Health-related quality of life (MOS-HIV)					
Physical health summary (PHS)	42.6	(11.0)	43.0	(10.7)	0.383
Mental health summary (MHS)*	44.0	(11.8)	45.6	(11.4)	0.001
General health perceptions	45.9	(10.2)	46.1	(10.0)	0.617
Physical functioning	45.8	(10.2)	46.0	(10.3)	0.654
Role functioning	41.3	(10.4)	41.4	(10.4)	0.838
Cognitive functioning*	42.0	(11.9)	43.8	(11.8)	0.001
Pain	47.6	(9.6)	48.0	(9.3)	0.288
Energy/fatigue	43.6	(10.6)	44.1	(10.4)	0.296
Mental health*	46.0	(11.6)	47.6	(11.6)	0.002
Health distress*	47.5	(11.8)	48.7	(11.5)	0.020
Social functioning*	42.5	(13.7)	43.7	(13.0)	0.057
Quality of life	44.9	(12.2)	45.7	(12.5)	0.199

*SD* standard deviation

* *p* values from McNemar (categorical) and paired student *t* test (continuous variables)

^a^ Includes individuals who are homeless (e.g., living on the street, cars, parks) or inadequately housed (e.g. living in hotels, motels, shelters, or couch-surfing

^b^ Very difficult or fairly difficult

HRQOL of participants improved slightly over the study period. The two summary measures, PHS and MHS, increased by 0.4 points and 1.6 points, respectively. However, only the increase in MHS was statistically significant (*p* < 0.05). Among the 10 MOS-HIV subscales, a statistically significant (*p* < 0.05) but modest increase was observed in cognitive functioning, mental health and health distress scores.

### Cross-Sectional Associations Between Housing Variables and HRQOL Measures

Regression analyses were conducted to examine the relationship between HRQOL (PHS and MHS) and demographic, HIV disease, psychosocial, and housing and neighborhood variables. Results of the regression models are presented in Table 3. Variables that were significantly associated (*p* < 0.05) with either physical or mental HRQOL (PHS or MHS, respectively) in bivariate regression analyses were entered in the multivariate models. Predictor variables were entered into the multivariate regression models in three blocks. Demographic and HIV disease variables were entered first. Psychosocial variables were entered into the models in the second block, followed by housing and neighborhood variables.

**Table 3:** Unadjusted and adjusted linear regression coefficients from cross-sectional analyses (*N* = 602)

Baseline predictors	Baseline physical health summary (PHS) score								Baseline mental health summary (MHS) score							
	Unadjusted (bivariate)		Adjusted (multivariate)						Unadjusted (bivariate)		Adjusted (multivariate)					
			Step 1		Step 2		Step 3				Step 1		Step 2		Step 3	
	β	*p*	β	*p*	β	*p*	β	*p*	β	*p*	β	*p*	β	*p*	β	*p*
Age (years)	−0.27	**<0.01**	−0.24	**<0.01**	−	**<0.01**	−0.25	**<0.01**	0.07	0.22	−0.04	0.51	−0.04	0.53	−0.10	0.07
Gender (female or transgender)	0.10	0.92	−1.63	0.11	−0.56	0.53	0.26	0.77	−3.80	**<0.01**	−4.07	**<0.01**	−4.30	**<0.01**	−2.91	**<0.01**
Ethnicity (Caucasian)	−3.18	**<0.01**	−2.35	**0.02**	−1.36	0.12	−1.40	0.11	−1.02	0.35	−2.58	**0.02**	−2.86	**<0.01**	−3.10	**<0.01**
Education (< high school)	−2.79	**<0.01**	−1.91	0.06	−1.55	0.08	−0.80	0.36	−3.71	**<0.01**	−1.89	0.10	−2.09	0.05	−1.47	0.15
Employment (employed)	6.97	**<0.01**	5.50	**<0.01**	3.85	**<0.01**	3.16	**<0.01**	5.98	**<0.01**	5.02	**<0.01**	3.87	**<0.01**	2.26	**0.03**
Alcohol use (AUDIT−10 score)	−0.01	0.86	0.00	0.99	0.06	0.36	0.04	0.45	−0.27	**<0.01**	−0.20	**0.01**	−0.21	**<0.01**	−0.17	**0.01**
Substance use (DAST−20 index)	−0.25	**<0.01**	−0.21	**0.02**	−0.09	0.23	−0.07	0.36	−0.38	**<0.01**	−0.23	**0.02**	−0.23	**0.01**	−0.24	**0.01**
Diagnosed with AIDS (yes)	−4.54	**<0.01**	−3.48	**<0.01**	−2.47	**<0.01**	−2.60	**<0.01**	−2.26	**0.02**	−2.50	**<0.01**	−2.77	**<0.01**	−2.65	**<0.01**
Years since HIV diagnosis (years)	−0.18	**<0.01**	0.01	0.89	−0.08	0.19	−0.09	0.17	0.26	**<0.01**	0.29	**<0.01**	0.28	**<0.01**	0.22	**<0.01**
Social support (MOS−SSS score)	0.12	**<0.01**			0.04	0.07	0.04	0.09	0.24	**<0.01**			0.23	**<0.01**	0.19	**<0.01**
Depressive symptoms (CESD−R score)	−5.89	**<0.01**			−5.29	**<0.01**	−4.73	**<0.01**	10.74	**<0.01**						
Live in the GTA (yes)	3.96	**<0.01**					2.28	**<0.01**	0.57	0.57					−0.20	0.83
Difficulty paying housing cost (yes)	−4.95	**<0.01**					−3.25	**<0.01**	−5.31	**<0.01**					−4.02	**<0.01**
Receive rental assistance (yes)	−2.85	**<0.01**					−0.80	0.314	−2.54	**0.01**					−1.35	0.14
Homeless/unstable housing (yes)	2.02	0.29					−0.26	0.89	−5.05	**<0.01**					2.67	0.23
Moved ≥2 times in the past year (yes)	−3.76	**<0.01**					−2.09	0.07	−4.11	0.05					−3.00	**0.02**
Dwelling features	0.78	0.08					0.71	0.09	1.81	**<0.01**					1.29	**<0.01**
Neighborhood characteristics	1.25	**<0.01**					0.52	0.19	2.38	**<0.01**					1.75	**<0.01**
Meaning of dwelling and neighborhood	−0.04	0.93					−0.51	0.19	2.61	**<0.01**					1.82	**<0.01**
Proximity to services and facilities	0.91	0.04					0.56	0.13	1.57	**<0.01**					1.23	**<0.01**
R^2^			0.157		0.348		0.397				0.131		0.263		0.345	
Adjusted R^2^			0.145		0.336		0.376				0.118		0.251		0.323	

All housing variables were included in the multivariate models. Control variables significantly (*p* < 0.05) associated with change in PHS and MHS only were included in the regression models. Depression was excluded from the MHS regression multivariate model due to multicollinearity

Bold indicates statistical significance (*p* < 0.05)

The multivariable regression model for physical HRQOL (PHS) showed that, among housing and neighborhood variables, living in the GTA was associated with a higher physical health (PHS) score; whereas difficulty paying housing cost was correlated with lower mental health (MHS) score. The association between physical health and receipt of rental assistance and housing instability (i.e., moving twice or more in the past 12 months) was not statistically significant. Similarly, none of the four housing satisfaction summary scores (i.e., ‘dwelling features’, ‘neighborhood characteristics’, ‘meaning of dwelling and neighborhood’, and ‘proximity to services and facilities’) were associated with physical health dimensions. Housing and neighborhood variables together accounted for 4.9 % of the total variation in the physical HRQOL summary score.

Among the control variables, younger age and being employed were significantly associated with higher baseline physical HRQOL, while having a diagnosis with at least one AIDS defining condition and higher depressive symptoms were associated with lower baseline physical HRQOL. Demographic and HIV disease variables accounted for 15.7 % of the variance in the model. The addition of psychosocial variables (i.e., social support and depressive symptoms) increased the variance of the model by 19.1 %. However, only depression was significantly associated with physical health score (β = −5.29).

In the multivariable regression model for mental HRQOL, baseline housing and neighborhood variables together accounted for 8.2 % of the variation in the model. After adjusting for other control variables, difficulty paying housing cost and housing instability were associated with lower mental HRQOL; whereas higher satisfaction with ‘dwelling features’, ‘neighborhood characteristics’, ‘meaning of dwelling and neighborhood’, and ‘proximity to services and facilities’ were associated with significantly higher mental HRQOL. Among the demographic variables, being female or transgender, having a Caucasian ethnicity, and higher levels of alcohol and illicit drug use were associated with lower mental HRQOL. HIV disease variables (i.e., diagnosis with AIDS defining condition and longer duration since HIV diagnosis) were also associated with lower mental HRQOL; whereas higher perceived social support was significantly associated with higher mental HRQOL. Demographic and HIV disease variables and perceived social support accounted for 13.1 and 13.2 % of the total variance in the model, respectively.

### Longitudinal Associations Between Housing Variables and HRQOL Measures

To examine the longitudinal association between housing and neighborhood variables and HRQOL measures, we fitted two multivariate regression models. The outcome variables were change in physical and mental HRQOL (PHS and MHS, respectively) between baseline and 1-year follow-up and baseline housing and neighborhood variables were the predictors. Sociodemographic, HIV disease, and psychosocial variables that were associated with change in physical and mental HRQOL in bivariate analyses were considered as control variables. In addition to these control variables, the models were also adjusted for baseline physical and mental HRQOL. Variables were entered into the regression models in three steps. Sociodemographic and HIV disease variables were entered as the first block. Perceived social support and baseline physical and mental HRQOL scores were entered as the second block. In the final and third block, housing and neighborhood variables were included in the model. Although depression was significantly associated with change in mental HRQOL, it was excluded from the multivariate regression models due to multicollinearity.

Results of the regression analyses are presented in Table 4. The models show that baseline housing and neighborhood variables together accounted for 1.3 and 4.1 % of the variance in changes in physical and mental HRQOL, respectively. Living in the GTA was associated with significant change both in physical and mental HRQOL over the 1-year period. Difficulty paying housing cost and two of the housing satisfaction measures (i.e., ‘neighborhood characteristics’ and ‘meaning of dwelling and neighborhood’) were also associated with significant changes in mental HRQOL score. Baseline physical and mental HRQOL scores significantly predicted change in physical and mental HRQOL scores. Age of participants at baseline predicted change in physical health, but not change in mental HRQOL scores.

**Table 4:** Unadjusted and adjusted linear regression coefficients from longitudinal analyses (*N* = 509)

Baseline predictors	Change in physical health summary (PHS) score Over 1-year follow-up								Change in mental health summary (MHS) score Over 1-year follow-up							
	Unadjusted (bivariate)		Adjusted (multivariate)						Unadjusted (bivariate)		Adjusted (multivariate)					
			Step 1		Step 2		Step 3				Step 1		Step 2		Step 3	
	β	*p*	β	*p*	β	*p*	β	*p*	β	*p*	β	*p*	β	*p*	β	*p*
Age (years)	−0.10	**0.04**	−0.10	**0.04**	−0.22	**<0.01**	−0.24	**<0.01**								
Years since HIV diagnosis (years)	−0.07	0.67							−0.15	**0.04**	−0.15	**0.04**	−0.02	0.79	−0.07	0.28
Depressive symptoms (CESD−R score)	2.00	**<0.01**			−0.93	0.05	−0.76	0.13								
Baseline PHS score	−0.37	**<0.01**			−0.44	**<0.01**	−0.46	**<0.01**								
Social support (MOS−SSS score)	−0.03	0.23							−0.08	**<0.01**			0.03	0.23	0.02	0.40
Baseline MHS score									−0.46	**<0.01**			−0.47	**<0.01**	−0.53	**<0.01**
Live in the GTA (yes)	0.19	0.81					1.57	**0.04**	1.97	**0.04**					2.89	**<0.01**
Difficulty paying housing cost (yes)	1.56	0.06					−0.71	0.35	0.74	0.45					−1.85	**0.04**
Receive rental assistance (yes)	0.71	0.39					−0.55	0.47	2.27	**0.02**					0.66	0.46
Moved twice or more in the past year (yes)	1.74	0.17					0.05	0.96	1.32	0.39					−0.87	0.52
Homeless/unstable housing (yes)	−1.17	0.58					−1.22	0.58	−1.87	0.46					0.33	0.90
Dwelling features	−0.38	0.34					0.30	0.44	0.01	0.99					0.83	0.08
Neighborhood characteristics	−0.46	0.25					0.11	0.79	−0.47	0.32					1.05	**0.02**
Meaning of dwelling and neighborhood	0.40	0.32					0.43	0.26	−0.40	0.42					0.92	**0.04**
Proximity to services and facilities	−0.58	0.15					−0.15	0.69	−0.48	0.32					0.29	0.51
R^2^			0.008		0.243		0.256				0.008		0.252		0.293	
Adjusted R^2^			0.006		0.238		0.238				0.006		0.247		0.276	

All housing variables were included in the multivariate models. Control variables significantly (*p* < 0.05) associated with change in PHS and MHS only were included in the regression models. Depression was excluded from the MHS regression multivariate model due to multicollinearity

Bold indicates statistical significance (*p* < 0.05)

## Discussion

We hypothesized that the housing and neighborhood characteristics of people living with HIV in Ontario would be associated with both physical and mental health-related quality of life (HRQOL). We found that living in the GTA and having less difficulty paying for housing cost were associated with higher physical HRQOL in the cross-sectional analysis, but only living in the GTA predicted significant improvement in physical HRQOL over the 1-year follow-up. We also found that greater difficulty paying for housing cost and moving twice or more in the past 12 months at baseline were associated with lower baseline mental HRQOL scores. However, in our longitudinal analysis, living in the GTA and having less difficulty paying for housing were significant predictors of improved mental HRQOL over time. Furthermore, baseline neighborhood characteristics and meaning of dwelling and neighborhood predicted improvement in mental HRQOL over the study period.

The association between residing in the GTA and physical and mental health-quality of life may be due to two reasons: greater access to health and social services and demographic differences. The better availability of or access to health and supportive services in the GTA compared to other Ontario communities may contribute to the differences in HRQOL. Our data shows, for example, that compared to people outside the GTA, a significantly higher proportion of those living in the GTA were more likely to have visited a family doctor, culturally appropriate services such as traditional healers, and dental care service providers in the 3 months period prior to the baseline interview. On the other hand, a significantly higher proportion of those outside of the GTA indicated the need for more access to services such as family doctor, HIV specialist, and home care nurse. It is also possible that the differences in HRQOL may be due to underlying demographic differences between those living in and outside the GTA. Participants from the GTA, for example, were younger and more educated. On the other hand, participants from outside the GTA were more likely to have a higher rate of history of incarceration and report higher level of alcohol and illicit drug use.

The finding that a greater difficulty paying for housing cost was associated with lower physical and mental HRQOL in cross-sectional analyses and the related decline in mental health over the 1-year follow-up period is consistent with the findings reported by Dunn and Hayes [34], who found an association between percentage of gross household income spent on housing costs and both self-reported general health and self-reported mental health. Higher housing cost may impact the well-being of individuals in two ways. First, higher housing cost relative to income can be a source of chronic stress [8]. The threat of losing housing due to increasing rent or mortgage payments can affect people’s health-related quality life negatively. Second, higher housing cost, particularly among those with fixed income, may restrict or reduce income that is available for other health-promoting approach, goods and lifestyle [7, 9]. For example, higher housing cost may lead to reduced expenditure on food, recreation, and personal care. People with HIV face high levels unemployment and poverty due to the disease itself [32, 44] and hence are more likely to experience difficulty paying for housing.

Our finding of a positive association between the meaning of dwelling and neighborhood and mental health is in agreement with previous research in the general population [34]. In a cross-sectional study, Dunn and Hayes [34] found a positive correlation between the meaning people invest in their homes, their satisfaction with their homes, and the amount of control they were able to exercise in the social and economic aspects of their domestic relations and their self-reported general health and mental health status. However, we examined this association further using longitudinal data and found that the meaning of dwelling and neighborhood was a significant predictor of improvement in mental HRQOL over time. These longitudinal associations suggest that the social meaning of one’s dwelling and neighborhood may contribute to mental well-being.

The positive association between a dwelling’s proximity to services and facilities and mental health is also plausible. Local availability of health-promoting amenities and access to health and social services may influence health [45]. To maintain or improve their health, most people living with HIV must receive regular treatment and monitoring by health care professionals. Therefore, good access or good transport link to health care providers may increase utilization of services. It is also possible that the lack or availability of recreational facilities in a neighborhood may influence the use of these amenities thereby contributing to good or bad health.

The results of this study should be considered in light of potential limitations. First, our study participants are primarily individuals affiliated with or receiving services from community-based AIDS service organizations, which limits the generalizability of our findings to those accessing these services. Individuals receiving services from community-based AIDS service organizations, for example, are more likely to report physical disability, difficulty sustaining normal activities, being depressed and poor HRQOL compared to those who do not receive services from these providers [46]. Second, participants who completed the 1-year follow-up were less likely to: be homeless or inadequately housed, report a history of homelessness, to move frequently, and report a history of incarceration at baseline than those who were lost-to-follow-up. Hence, our results from the longitudinal analyses may not be generalizable to all participants enrolled in our study. The attrition may also have led to underestimation of the effect size in our longitudinal analyses. Third, our sample has a small number of individuals who were homeless or inadequately housed and hence, our findings may not reflect the experiences of those who are the most vulnerable. Fourth, all data including housing and HIV disease variables are self-reported and were collected through face-to-face interviews. As such, data may be subject to socially desirable response biases.

Despite its limitations, our study is the first to examine the associations between the material, meaningful and spatial dimensions of housing and both physical and mental HRQOL of people living with HIV in Canada. Our study also demonstrated that influence of housing dimensions on both physical and psychological HRQOL longitudinally, while controlling for an important and comprehensive set of covariates and baseline HRQOL.

Our findings add to the understanding about housing variables that predict changes in health (both physical and mental) related quality of life over a 1-year period. The findings also have implications for interventions and future research. First, difficulty paying housing costs has a significant detrimental effect on HRQOL. As most people with HIV live on the economic margins, lack of affordable housing can easily trap them in inappropriate, unsuitable and unhealthy housing. Difficulty paying for housing may make people vulnerable to a forced move or eviction. Nearly a third of our study participants worry that they may be forced to move out of their housing. Living in unsuitable housing with the constant threat of losing control over housing may negatively impact physical and mental well-being through stress-related mechanisms. Therefore, it is likely that increasing availability of affordable housing would help improve quality of life for people with HIV. Second, our findings about meaningful dimensions of housing and neighborhoods emphasize the importance of portable rental assistance programs that would allow individuals to find housing in neighborhoods or communities of their choice. Living in low-quality and run-down housing in neighborhoods reputed to be less desirable may exert considerable influence on how individuals perceive themselves and the way they are perceived by others [7]. The stigma associated with one’s residence, therefore, may undermine the home’s social meaning and its use as a site for the building of social ties within a community. When individuals are able to obtain housing that increases social ties and meaningful dimensions, it is likely that their physical and mental HRQOL will also improve.

## Footnotes

1 Compared to the 93 participants lost to follow-up, the 509 individuals who remained in the current study had been infected with HIV longer (11.5 vs. 9.5 years, *p* < 0.01), were more likely to be on antiretroviral treatment (77 vs. 59 %, *p* < 0.01), and reported lower illicit drug use (mean DAST-20 score: 3.8 vs. 5.3, *p* < 0.01), lower burden of depression (mean CESD-R score: 17.3 vs. 21.2, *p* < 0.05), and higher perceived social support (mean MOS-SSS score: 62.8 vs. 58.2, *p* < 0.05) at baseline. Included participants also were less likely to: live in the GTA (59 vs. 80 %, *p* < 0.01), be homeless or inadequately housed (4 vs. 16 %, *p* < 0.01), report a history of homelessness (40 vs. 56 %, *p* < 0.01), have moved twice or more in the past 12 months (11 vs. 18 %, *p* < 0.05), and have a history of incarceration (29 vs. 44 %, *p* < 0.01). They also were more satisfied with various dimensions of their housing and neighborhood.
